# Glucocorticoid Receptor and Adipocyte Biology

**DOI:** 10.32527/2018/101373

**Published:** 2018

**Authors:** Rebecca A. Lee, Charles A. Harris, Jen-Chywan Wang

**Affiliations:** 1 Endocrinology Graduate Program and Department of Nutritional Science & Toxicology, University of California Berkeley, Berkeley, CA 94720-3104, USA; 2 Division of Endocrinology, Metabolism, and Lipid Research, Washington University School of Medicine, St. Louis, Missouri 63110, USA

**Keywords:** Glucocorticoid Receptor, Adipocyte, White Adipose Tissue, Brown Adipose Tissue

## Abstract

Glucocorticoids are steroid hormones that play a key role in metabolic adaptations during stress, such as fasting and starvation, in order to maintain plasma glucose levels. Excess and chronic glucocorticoid exposure, however, causes metabolic syndrome including insulin resistance, dyslipidemia, and hyperglycemia. Studies in animal models of metabolic disorders frequently demonstrate that suppressing glucocorticoid signaling improves insulin sensitivity and metabolic profiles. Glucocorticoids convey their signals through an intracellular glucocorticoid receptor (GR), which is a transcriptional regulator. The adipocyte is one cell type that contributes to whole body metabolic homeostasis under the influence of GR. Glucocorticoids’ functions on adipose tissues are complex. Depending on various physiological or pathophysiological states as well as distinct fat depots, glucocorticoids can either increase or decrease lipid storage in adipose tissues. In rodents, glucocorticoids have been shown to reduce the thermogenic activity of brown adipocytes. However, in human acute glucocorticoid exposure, glucocorticoids act to promote thermogenesis. In this article, we will review the recent studies on the mechanisms underlying the complex metabolic functions of GR in adipocytes. These include studies of the metabolic outcomes of adipocyte specific GR knockout mice and identification of novel GR primary target genes that mediate glucocorticoid action in adipocytes.

## 1. Introduction

Glucocorticoids are steroid hormones that convey their signals through the intracellular glucocorticoid receptor (GR), which is a transcriptional regulator. Before binding to hormones or ligands, the majority of GR is localized in the cytosol and associates with the heat shock protein 90 (hsp90)-containing chaperone complex [1–4]. Upon binding to hormones or ligands, the heterocomplex of GR-hsp90-containing chaperone complex can passage through the nuclear pore [1–4]. In the nucleoplasmic region, GR is dissociated from hsp90-containing chaperone complex and associates with genomic glucocorticoid response elements (GREs) to regulate the transcriptional rate of nearby genes. GR usually binds to GREs as a homodimer, though it could also modulate the transcription as a monomer through binding to the DNA [5] or interaction with other transcription factors [6]. Glucocorticoids play critical roles in the regulation of metabolic homeostasis. Upon stress, such as fasting and starvation, glucocorticoids exert their metabolic functions through multiple cell types to maintain plasma glucose levels, which is the major energy source for brain. Excess and/or chronic glucocorticoid exposure, however, causes metabolic disorders that include insulin resistance, hyperglycemia, dyslipidemia, and central obesity. Glucocorticoid excess does not need to be systemic to result in metabolic syndrome. In fact, patients with metabolic syndrome do not have elevated glucocorticoid levels but are thought to have elevated local levels of glucocorticoids in adipose tissue. This is possible due to local activation of glucocorticoids in adipose tissue by the enzyme 11β-hydroxysteroid dehydrogenase type 1 (11β-HSD1), which encodes an enzyme that converts the inactive glucocorticoid cortisone to active the glucocorticoid cortisol or corticosterone [7, 8]. Interestingly, transgenic expression of 11β-HSD1 limited to adipose tissue, results in mice with metabolic syndrome while fed a chow diet [9]. In contrast, overexpression of 11β-HSD2 (which inactivates glucocorticoids) in adipose tissue protects mice from metabolic complications of diet induced obesity [10]. Inhibitors of 11β-HSD1, have been in clinical trials for treating metabolic syndrome [7, 8, 11]. There are some adverse effects reported for 11β-HSD1 inhibitors which include the compensatory increase of hypothalamus-pituitary-adrenal (HPA) axis and an elevated androgen production [11, 12]. It is important to note that reducing glucocorticoid signaling in animal models of metabolic disorders usually improves their metabolic profiles and insulin sensitivity [13–15]. However, not surprisingly, reducing glucocorticoid signaling universally would result in undesired adverse effects.

Adipose tissue plays a key role in whole body metabolic homeostasis. Glucocorticoids affect multiple aspects of adipocyte biology. First, glucocorticoids are included in almost all differentiation media for adipogenesis in vitro. However, the exact role of glucocorticoids and GR in adipose tissue development *in vivo* has not been established. Second, glucocorticoids exert complex effects on lipid metabolism in adipose tissues. Upon fasting, glucocorticoids increase lipolysis in white adipose tissues, generating glycerol and fatty acids. The former is the precursor for hepatic gluconeogenesis whereas the latter is an energy source during fasting [16, 17]. However, in pathological states, glucocorticoid effects on lipid metabolism in adipose tissue are more complicated. Patients with Cushing’s syndrome, which is characterized by chronically elevated circulating cortisol levels, develop central obesity, but subcutaneous white adipose tissue is reduced, resulting in a lipodystrophy [18, 19]. The molecular basis of these fat depot specific effects of glucocorticoids is unclear. One possibility is depot-specific balance between lipogenic and lipolytic gene expression, which has been investigated in human visceral and subcutaneous adipose derived stem cells [20] as well as in adipose tissue harvested from obese individuals [21]. In fact, in rodents, chronic glucocorticoid exposure augments adiposity but is usually not restricted to visceral fat depots [22]. Third, glucocorticoids suppress insulin-stimulated glucose uptake in adipocytes, which might contribute to the modulation of whole body glucose homeostasis and insulin sensitivity [23–25]. The mechanism underlying glucocorticoid-induced insulin resistance in adipocytes is not entirely clear. Notably, certain studies find that glucocorticoids enhance, rather than suppress, insulin actions in human primary adipocytes [26, 27]. Finally, glucocorticoids have been shown to repress the thermogenic activity of brown and beige adipocytes in rodents [28–31]. However, recent studies show that acute glucocorticoid exposure enhances the thermogenic activity of human brown adipose tissue [32]. The reason for such species-specific effects is unknown. Studies over the last several years have made significant contributions to our understanding of glucocorticoid functions in various aspects of adipocyte biology. We will review and discuss these new developments below.

## 2. The Role of Glucocorticoids and GR in Adipogenesis *in vivo*

Glucocorticoids are included in most differentiation protocols that are used to induce adipogenesis in vitro. Glucocorticoids have been shown to promote preadipocytes (3T3-L1 and 10T1/2 cells) to an intermediate priming state before committing them to differentiation [33]. GR has been shown to act with other adipogenic transcription factors, such as C/EBPβ, to activate the transcription of PPARγ2, a master regulator of adipogenesis [34]. Transcriptional coregulators of GR [35] and some GR primary target genes are found to be required for adipocyte differentiation in vitro [36–38]. However, the exact role of GR in adipose tissue development had not been explored until two recent publications. GR^*flox/flox*^ mice were crossed with mice expressing Cre recombinase under the control of Myf5 promoter to delete the GR gene in brown adipocyte precursor cells [39]. There was no apparent difference in brown adipose tissue development between these mice and wild type mice. This suggests that GR is not required for brown adipose tissue development *in vivo*. Preadipocytes isolated from brown adipose tissue of brown fat specific GR knockout mice showed immature adipocytes at day seven of differentiation compared to those differentiated from preadipocytes of wild type mice. However, the number of mature adipocytes were comparable at day 14 and day 21 of differentiation [39]. Similar results were observed in preadipocytes isolated from inguinal white adipose tissue of GR^*flox/flox*^ mice that were infected with adenoviruss expressing GFP or Cre recombinase [39]. These results demonstrate that GR is dispensable but can accelerate both white and brown adipocyte differentiation.

In another study, several approaches were used to test the role of GR and endogenous glucocorticoids in adipose tissue development. First, mouse embryonic fibroblasts (MEFs) isolated from GR knockout mice (by crossing GR^*flox/flox*^ with *actin*−Cre mice) and wild type mice were both capable of forming *de novo* fat pads in mice [40]. However, GR-null fat pads and their associated adipocyte areas were smaller than those in controls [40]. Second, eliminating circulating corticosterone by adrenalectomy does not block the formation of *de novo* fat pads in mice, though the fat pads formed in adrenalectomized mice were smaller than those in control mice [40]. Moreover, using adipocyte-specific luciferase reporter, mouse adipocytes were identified in day 18 mouse embryos in both WT and GR-null mice [40]. Positive perilipin staining was also identified in day 18 embryos of WT and GR-null mice, which confirms the presence of early white inguinal and brown adipocytes [40]. Overall, these *in vivo* studies indicate that while GR and glucocorticoids can promote adipocyte differentiation, they are not required for the development of brown and white adipose tissues.

## 3. Regulation of Lipid Metabolism by GR in White Adipocytes

As discussed above, patients with Cushing’s syndrome have increased visceral fat depots but decreased subcutaneous fat depots. This suggests that glucocorticoids can modulate both lipogenesis and lipolysis in white adipose tissue. Exogenous glucocorticoid treatment has been shown to stimulate both triglyceride synthesis and lipolysis in white adipose tissue [41]. Similarly, using stable isotope labeling techniques, it was found that de novo lipogenesis, triglyceride synthesis and lipolysis were all concurrently stimulated in white adipose tissues of corticotropin-releasing hormone overexpressing mice [22]. Thus, it is possible that other environmental factors in distinct fat depots affect the ability of glucocorticoids to modulate either lipogenesis and/or lipolysis. The change in the ratio of the rate of lipogenesis vs. lipolysis will result in alteration of adiposity. This notion, however, has not been examined.

**Figure 1:**
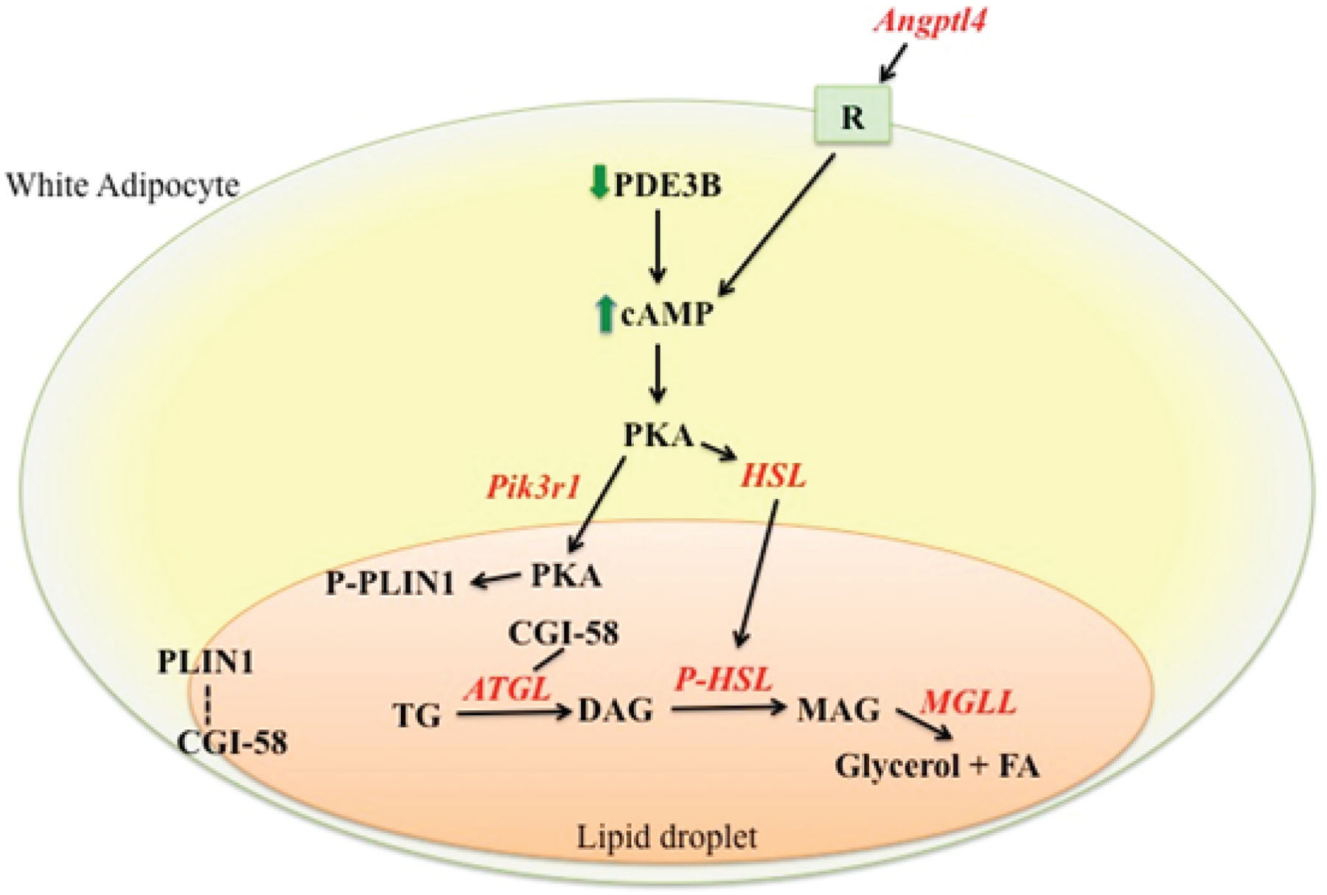
The mechanism of glucocorticoid-induced lipolysis in adipocytes. Glucocorticoids activate several mechanisms to promote lipolysis in adipocytes. First, glucocorticoids decrease the expression of Pde3b, which results in the activation of cAMP signaling. Second, a list of lipolytic genes, *Atgl*, *Hsl*, and *Mgll*, are transcriptionally activated by GR. *Angptl4* is another GR target gene, which encodes a secreted protein that binds to an unidentified receptor to stimulate cAMP production to activate lipolysis. *Pik3r1* is an additional GR primary target gene that encodes a protein required for increasing PKA levels in the lipid droplet upon glucocorticoid treatment. Phosphorylation of Plin1 by PKA in the lipid droplet allows CGI-58 to dissociate from Plin1 and serves as an Atgl coactivator to promote lipolysis.

Various reports have shown that glucocorticoid treatment on differentiated adipocytes enhances lipolysis [42, 43]. This response, however, requires a much longer treatment time than that of catecholamine. This is consistent with the fact that glucocorticoids mainly modulate gene expression levels, which need to be translated to proteins to affect cellular physiology. Glucocorticoids stimulate lipolysis in adipocytes through multiple mechanisms. It has been shown that glucocorticoid treatment decreases the expression of cyclic-nucleotide phosphodiesterase 3B (Pde3b) in adipocytes, which results in elevated cAMP levels in adipocytes that potentiate lipolysis by phosphorylating hormone sensitive lipase (Hsl, a.k.a Lipe) and perilipin 1 (Figure 1) [42]. Notably, there is no GR binding site identified in or nearby the *Pde3b* gene locus. Thus, *Pde3b* may not be a primary target gene of GR. In contrast, glucocorticoids have been shown to activate the expression of genes encoding enzymes in the lipolytic pathway, such as hormone sensitive lipase (*Lipe)* [41], adipocyte triglyceride lipase (*Atgl*, a.k.a *Pnpla2*) [44] and monoglyceride lipase (*Mgll*) (Figure 1) [41]. All these three genes are potential GR primary target genes, as they contain GR binding regions in their genome. It has been shown that *Atgl* [45, 46] and *Lipe* [46] expression is significantly decreased in adipocyte specific GR knock out mice. However, the activity of these enzymes was not monitored. Therefore, the exact contribution of the induction of these lipolytic genes in glucocorticoid-promoted lipolysis *in vivo* is unclear. Notably, several GR primary target genes have been shown to be involved in glucocorticoid-induced adipose tissue lipolysis *in vivo*. *Angiopoietin-like 4* (*Angptl4*) is a GR primary target gene in hepatocytes and adipocytes that encodes a secreted protein [47]. The ability of exogenous glucocorticoids to promote adipose tissue lipolysis is reduced in *Angptl4* null mice (*Angptl4*^−/−^*)* [47]. Glucocorticoid treatment elevates the levels of Hsl and perilipin 1 phosphorylation in white adipose tissue of wild type mice but these effects are attenuated in *Angptl4*^−/−^ mice [47]. Purified human ANGPTL4 protein is able to directly induce cAMP levels in mouse primary adipocytes to promote lipolysis (Figure 1) [47]. *Phosphatidylinositol 3-kinase regulatory subunit 1* (*Pik3r1*) is another GR primary target gene in adipocytes and myotubes [48]. Exogenous glucocorticoid-induced adipose tissue lipolysis is reduced in adipocyte specific *Pik3r1* knockout mice [49]. Interestingly, in these mice, glucocorticoids still enhance the phosphorylation of Hsl but not perilipin 1 in white adipose tissue [49]. Because Hsl is localized in cytosol whereas perilipin 1 is localized on lipid droplets before phosphorylation, this data suggests that in *Pik3r1* null adipocytes, glucocorticoid activated protein kinase A (PKA) is specifically impaired in the lipid droplet. Indeed, in white adipose tissue of wild type mice, glucocorticoid treatment increases levels of both the catalytic and regulatory subunit of PKA on lipid droplets. But this effect is attenuated in adipocyte specific Pik3r1 knockout mice (Figure 1) [49]. Finally, modulating the phosphorylation status of GR could affect its ability to regulate lipid metabolism. Protein phosphatase 5 (PP5) is a component of hsp90-containing chaperone complex and can dephosphorylate GR through its phosphatase activity [50–53]. Mouse embryonic fibroblasts (MEF) lacking PP5 showed increased phosphorylation of GR at serine 212 and 234, and enhanced dexamethasone effect on lipolytic gene expression [54]. The ability of PP5 knockout MEFs to differentiate adipocyte is also significantly impaired. This phenotype, however, might not be entirely attributed to the regulation of GR activity by PP5, as PP5 also regulates PPARγ activity [54].

In contrast to lipolysis, the effect of glucocorticoids on lipogenesis in adipocytes is much more complicated. Glucocorticoids decrease lipogenesis in human adipocyte cell line Chub-S7 [55]. Similarly, in adipocytes differentiated from human primary subcutaneous and omen-tal preadipocytes, glucocorticoids also decrease lipogenesis [55]. However, in adipocytes also treated with insulin, glucocorticoids either enhance insulin-stimulated lipogenesis or, at the very least, do not suppress lipogenesis. [55]. Glucocorticoid treatment has been previously shown to increase the expression of certain lipogenic and triglyceride synthetic genes, such as *fatty acid synthase* (*Fasn*), *acetyl-CoA carboxylase 2* (*Acacb*), *glycerol-3-phosphate acyltransferase 3* and *4* (*Gpat3* and *Gpat4*) and *lipin 1* (*Lpin1*) in adipocytes, and some of these genes are direct target genes of GR [41, 55]. It appears that the induction of these genes by glucocorticoids is not able to promote lipogenesis. It is likely that other factors, such as insulin, are needed to act with glucocorticoids to enhance lipogenesis. Notably, upon insulin resistance, while insulin fails to exert its effects on glucose homeostasis, insulin still is able to enhance lipogenesis [56]. Thus, it is conceivable that during a state of insulin resistance, glucocorticoids act with insulin to further promote lipogenesis.

*FoxA3*, which encodes a transcription factor, has been identified as a primary target gene of GR in adipocytes [57]. Reduction of FoxA3 expression in adipocytes resulted in deregulation of more than 60% of glucocorticoid-regulated lipid metabolism genes, such as *Lipin1*, *Gpat4*, *Angptl4*, and *Lipe* [57]. Additionally, they found that in adipocytes, Foxa3 deficiency suppressed GR-induced lipolysis, but cAMP or PPARα-agonist–induced lipolysis was unaffected [57]. Chromatin immunoprecipitation experiments found that reducing FoxA3 expression attenuated the recruitment of GR to its genomic response elements [57]. In *FoxA3* null mice, exogenous glucocorticoid treatment-increased adiposity is compromised; however other glucocorticoid-promoted metabolic disorders, such as hepatic steatosis, were not affected [57]. The ability of glucocorticoids to induce lipid metabolism genes is also attenuated in adipose tissues but not in the liver of *FoxA3* null mice [57]. Thus, FoxA3 mediates and modulates GR actions specifically in adipocytes.

## 4. Regulation of Glucose Metabolism by GR in White Adipocytes

Glucocorticoids have frequently been shown to inhibit insulin-stimulated glucose uptake and utilization in adipocytes [25, 58, 59]. One mechanism by which glucocorticoids act to suppress insulin stimulated glucose uptake in 3T3-L1 mature adipocytes is to augment the production of reactive oxygen species (ROS) [60]. Treating 3T3-L1 adipocytes with anti-oxidants or overex-pressing genes encoding enzymes that can reduce ROS levels improved glucocorticoid-induced insulin resistance [60]. Treating genetically insulin resistant ob/ob mice with anti-oxidant, MnTBAP, significantly improved glucose and insulin tolerance [60]. Notably, ob/ob mice have elevated plasma corticosterone levels [61]. TNFα (acting through NFκB, a heterodimer of p65 and p50) also induces insulin resistance by elevating ROS in 3T3-L1 adipocytes [60]. Based on these observations, Kang et al. hypothesized that there are common “insulin resistance-induced genes” that are regulated by glucocorticoids and TNFα independently. Transcriptional profiling was performed and found that 271 dexamethasone-induced genes whose expression is also stimulated by TNFα treatment [62]. Kang et al. then performed chromatin immunoprecipitation sequencing of acetylated histone H3 lysine 27 (H3K27ac) to identify enhancers that are activated by dexamethasone or TNFα treatment. Within 200 kb of these 271 glucocorticoidand TNFα-induced genes, 326 enhancers were induced by glucocorticoid treatment whereas 598 enhancers were induced by TNFα treatment, but only 53 enhancers were found in both groups [62]. Bioinformatic analysis found that in addition to GRE, vitamin D receptor response element is highly presented in these 53 enhancers [62]. Indeed, reducing the expression of vitamin D receptor (VDR) in 3T3-L1 adipocytes reduces the ability of glucocorticoids and TNFα to inhibit insulin–stimulated glucose uptake [62]. VDR is a potential GR primary target gene, as it contains GR binding sites in its genomic region and its expression is induced by glucocorticoids [41, 62]. Notably, it seems that vitamin D, the ligand of VDR, is not required for the role of VDR in glucocorticoid and TNFα induced insulin resistance [62]. Another mechanism that might mediate glucocorticoid-induced insulin resistance is the induction of Mkp-1 (a.k.a Dusp1) by glucocorticoids [63]. Mkp-1 is a GR primary target gene containing functional GR binding sites [64, 65] that encodes a protein phosphatase mainly for p38 MAP kinase. Glucocorticoid treatment reduced insulin-induced p38 activation in 3T3-L1 adipocytes [63]. Moreover, overexpression of Mkp-1 in 3T3-L1 adipocytes mimicked the inhibitory response of glucocorticoids on insulin-stimulated glucose uptake [63]. Inhibition p38 MAP kinase has been shown to reduce the maximal induction of insulin-stimulated glucose uptake in adipocytes in some reports [66, 67]. However, in some other reports, inhibition of p38 MAP kinase does not affect insulin-stimulated glucose uptake in adipocytes [68–70]. The role of p38 MAP kinase inhibition in the suppressive effect of Mkp-1 overexpression on insulin-stimulated glucose uptake was not examined in this report. Furthermore, the role of adipocyte Mkp-1 in glucocorticoid effects on insulin sensitivity has not been tested *in vivo*. Thus, the exact role of Mkp-1 in glucocorticoid response in adipocytes requires more extensive studies.

Glucocorticoids have also been shown to modulate the expression of several adipokines that include plasminogen activator inhibitor-1 (PAI-1, a.k.a Serpine1), lipocalin 2 (Lcn2), and Angptl4. *PAI-1* is a GR primary target gene [71]. It has been shown that exogenous corticosterone treatment strongly elevated the expression of PAI-1 in circulation and white adipose tissues [72]. In both male and female *PAI-1* null mice, exogenous corticosterone treatment-induced glucose and insulin intolerance were compromised [72]. However, glucocorticoid-induced hyperlipidemia was not affected in PAI-1 null mice [72]. Treating human HepG2 hepatoma cells with PAI-1 reduced insulin signaling and the suppressive effect of insulin on gluconeogenic gene expression. In contrast, PAI-1 did not affect insulin signaling in 3T3-L1 adipocytes and C2C12 myotubes. These results suggest that the main target tissue for PAI-1-induced insulin resistance is liver [72]. The molecular mechanism governing the inhibitory effect of PAI-1 on insulin signaling in hepatocytes, however, is largely unclear. Glucocorticoid-induced glucose intolerance and insulin resistance was compromised in *Angptl4*^−/−^ mice [73]. In wild type mice, exogenous glucocorticoid treatment stimulated the hepatic ceramide synthetic program, which activated protein kinase c ζ (PKCζ) and protein phosphatase 2 A (PP2A) to inhibit insulin action in liver [73]. Without Angptl4, however, hepatic ceramide production induced by glucocorticoids was significantly attenuated [73]. Therefore, glucose intolerance and insulin resistance were improved in exogenous glucocorticoid treated *Angptl4*^−/−^ mice [73]. Reducing PKCζ and PP2A activity both attenuated the ability of glucocorticoids to induce insulin resistance and glucose intolerance [73]. *Lcn2* is another GR primary target gene [74, 75]. *Lcn2* null mice were partially protected from high fat diet induced glucose intolerance and insulin resistance [76, 77]. However, its role in exogenous glucocorticoid-induced insulin resistance has not been tested.

Notably, there are reports showing that while glucocorticoid treatment induces insulin resistance in primary adipocytes isolated from human omental white adipose tissue, it actually enhances insulin action in human primary subcutaneous adipocytes [26, 27]. The molecular basis of this depot specific effect on human primary adipocytes is unknown.

## 5. TheRole of Adipocyte GR in the Regulation of Metabolic Homeostasis *in vivo*

To analyze the exact role of GR in adipocytes in the regulation of metabolic homeostasis, several groups have created adipocyte specific GR knockout mice (AGRKO). The results from AGRKO mice are somewhat different between different laboratories (see Table 1). Most studies did not find any difference in adiposity in mice fed with chow diet. Most studies also did not observe any difference in glucose and insulin tolerance between wild type and AGRKO mice when fed with chow diet [45, 46, 78]. However, one report found that AGRKO mice are more insulin tolerant [79]. Additionally, they found the activity of AKT, a downstream effector of insulin signaling, is higher in the liver of AGRKO mice [79]. AGRKO mice had similar *Hsl* mRNA levels after a 48hr fast, while *Atg*l and *CgI-58* expression were reduced in AGRKO mice [79], while another group found *Plin1* and *Hsl* expression to be reduced in AGRKO mice [45]. They also found that fasting-induced adipose tissue lipolysis and hepatic gluconeogenesis are both reduced in AGRKO mice [79]. The role of adipocyte GR in adipose tissue lipolysis is supported by two reports. Injecting isoproterenol into wild type mice resulted in elevated plasma non-esterified fatty acids (NEFA) and glycerol, indicating an increase in adipose tissue lipolysis. This effect is decreased in AGRKO mice [45]. Both basal and isoproterenol-stimulated lipolysis were lower in fat explants isolated from AGRKO mice than those of wild type mice [45]. Moreover, 48 hour fasting-induced lipolysis was impaired in AGRKO mice [79]. This is likely due to the impairment of β-adrenergic receptor signaling in adipose tissue of AGRKO mice [79]. PKA activity in epididymal white adipose tissue explants was lower in 48 hour fasted AGRKO than wild type mice [79]. Glucocorticoid treatment increased the expression of *Gsα* (a.k.a *Gnas*) in white adipose tissue but not in liver and skeletal muscle. This induction was diminished in AGRKO mice [79]. Notably, adipocyte specific *Gsα* null mice also have defective adipose tissue lipolysis [80]. Whether *Gsα* is a GR primary target is unknown.

Two groups investigated a potential compensatory role of mineralocorticoid receptor (MR) [81] in the AGRKO mice. This question is relevant because cortisol/corticosterone efficiently bind to and can act through MR. One group found MR mRNA levels in adipose to increase slightly, but not significantly [78], while another group found no change in MR expression under high fat feeding in AGRKO mice [45]. Additionally, one group examined the effects of corticosterone in AGRKO mice [46]. They found that corticosterone treatment produced no significant difference in glucose tolerance and insulin sensitivity between wild type and AGRKO mice [46]. Notably, comparing to wild type mice, adipocyte specific MR knock out mice also showed no significant differences in adipose tissue weight or size, and glucose and insulin tolerance under high fat diet feeding [82]. Interestingly, overexpressing MR specifically in adipocytes has been shown to increase body weight, fat mass, and insulin resistance in mice [83]. Thus, while adipocyte MR might play a minor role in glucocorticoid- and high fat diet-induced metabolic disorders, increasing MR activity in adipocytes causes negative impacts on metabolic homeostasis. Previous reports have shown that treating high fat diet fed mice with MR antagonists induced the beiging of both visceral and inguinal white adipose tissue and improved glucose tolerance and adipose tissue dysfunction by reducing reactive oxygen species production and inflammatory process [84, 85]. Based on adipocyte specific MR knockout studies, the phenotypes observed in these reports could be due to the effects of MR antagonists on tissues other than adipose tissues.

Two groups looked at hypothalamus-pituitary-adrenal (HPA) axis function in AGRKO mice. In one study, compared to wild type mice, acute 30 minute restraint, a psychogenic stressor, causes hypersecretion of corticosterone and hyperglycemia in AGRKO mice [86]. Injecting low dose dexamethasone for 2 hours prior to the restrain t showed that AGRKO mice are able to escape from dexamethasone suppression faster than wild type mice [86]. Another study performed a dexamethasone suppression test, looking at corticosterone levels before and 4 hours after a 1mg/kg dose of dexamethasone. This study found equal suppression of corticosterone in AGRKO vs. wild type mice [46]. A possible reason for the discrepant data is an influence of adipose GR on the stress response vs. direct pharmacologic treatment. It is not clear how adipose GR would influence the HPA axis. Another possible explanation could be a developmental compensation arising during development. Finally, it is also possible that there exists a circuit between adipose sensory nerves and the hypothalamus.

Under high fat diet feeding, three reports did not observe significant differences in adiposity, as well as glucose and insulin tolerance between wild type and AGRKO mice [45, 46, 78]. However, the other two reports found that AGRKO mice gain weight slower than wild type mice [79, 86]. Moreover, glucose tolerance was found to be improved in AGRKO mice [79]. Plasma insulin levels were lower and insulin-induced AKT activity were improved in AGRKO mice [79]. High fat diet-induced hepatic steatosis and lipogenic gene expression were also reduced in AGRKO mice [79]. In addition to high fat diet feeding, aging-induced body weight gain and hepatic steatosis were also compromised in AGRKO mice in this report [79]. High fat diet induced inflammation in epididymal white adipose tissue was enhanced in AGRKO mice [78]. These results are in agreement with the anti-inflammatory functions of GR and previous observations show how glucocorticoid treatment, despite causing insulin resistance, attenuates high fat diet induced macrophage recruitment into white adipose tissue [87].

The response of AGRKO mice to exogenous glucocorticoid-induced insulin resistance also differed between different studies. In one study, exogenous glucocorticoid-induced glucose and insulin intolerance were improved in AGRKO mice [45]. Glucocorticoids inhibit insulin-stimulated Akt activity in the soleus muscle of wild type mice, which was also compromised in AGRKO mice [45]. In another study, wild type and AGRKO mice were treated with either synthetic glucocorticoid dexamethasone or physiological glucocorticoid corticosterone. However, no significant difference in adiposity, glucose and insulin tolerance were observed between wild type and AGRKO mice [46]. One difference between these two studies is that one study treated mice with dexamethasone for 2 months (2mg/kg body weight/day) [45], whereas the other study treated mice with dexamethasone for 2 weeks (10mg/kg body weight/day) or corticosterone (10mg/kg body weight/day) for 4 weeks [46]. Whether the difference in duration and dose of ligand treatment can result in distinct outcomes is unclear.

Notably, among these 5 studies, 32 of them used the same GR-flox mice generated by the Schütz lab [88] (Table 1). In contrast, 2 used GR-flox mice generated from the Ashwell lab [89] and de Kloet et al. used GR-flox mice generated from the Muglia lab [90] (Table 1). Nonetheless, sometimes, different laboratories observe distinct metabolic outcomes even for the same transgenic or knockout mouse lines. Multiple studies on AGRKO mice, thus, are valuable. We do not know the exact reasons for the differential observations between these studies. However, the overall results demonstrate that adipocyte GR plays a role in whole body metabolic homeostasis *in vivo*. This role may appear less obvious in AGRKO mice if developmental compensation occurs. The robust difference in the serum metabolome described in one study is potential evidence for such compensation [91].

## 6. The Role of GR in the Regulation of Brown and Beige Adipocyte Function

It has been shown that glucocorticoid treatment in rodents decreases thermogenic activity of BAT and suppresses beiging. In rodents, glucocorticoids have been shown to reduce the expression of uncoupling protein 1 (Ucp1) [29, 92], which encodes a protein that is specifically expressed in brown and beige adipocytes required to generate heat during adaptive thermogenesis [93, 94]. Moreover, adrenalectomy and GR antagonist increased the expression of Ucp1 and elevates BAT thermogenesis [28, 95, 96]. Similarly, mice lacking 11β-hydroxysteroid dehydrogenase type 1 (*11β-HSD1*^−/−^) were protected by suppression of BAT activity by exogenous glucocorticoids [97]. Although the expression of Ucp1 was similar between corticosterone treated wild type and *11β-HSD1*^−/−^ mice, several genes encoding proteins in the activation of thermogenesis and mitochondrial respiration were expressed in lower levels in *11β-HSD1*^−/−^ mice [97]. Treating primary brown adipocytes isolated from wild type mice with corticosterone reduced oxygen consumption and the expression of thermogenic genes, such as Ucp1, and brown/beige makers Cox8b and Cox7a1, which encodes proteins involved in mitochondrial oxidative phosphorylation [97]. Interestingly, although corticosterone still suppressed the expression of thermogenic and mitochondrial genes in primary brown adipocytes isolated from *11β-HSD1*^−/−^ mice, the basal expression of these genes was higher than those of wild type mice [97]. Similarly, the oxygen consumption rate was also higher in primary brown adipocytes of *11β-HSD1*^−/−^ mice [97]. These results suggest that the glucocorticoid effect on thermogenesis is cell autonomous. Notably, lysine-specific demethylase 1 (Lsd1) promotes BAT thermogenesis by inhibiting *11β-HSD1* expression in BAT [98]. Adipocyte specific *Lsd1* deletion impaired mitochondrial fatty acid oxidation capacity of BAT, reduced whole-body energy expenditure, and increased fat deposition [98]. These phenotypes, however, were alleviated by deleting *11β-HSD1* concurrently [98]. Overall, glucocorticoids appear to have species-specific effects on BAT activation. In humans, glucocorticoid treatment has been shown to increase BAT activity and UCP-1 expression [32, 99]. However, chronic glucocorticoid treatment in human adipocytes did decrease UCP-1 expression after 48hrs of treatment [32].

AGRKO mice are generated by crossing *GR*^*flox/flox*^ mice with mice expressing Cre recom binase driven by an adiponectin promoter. Since adiponectin is expressed in both white and brown adipose tissue, GR is also deleted in the brown adipocytes of these mice. When wild type and AGRKO mice were fasted for 4 hours, and then placed in 4°C for 4 hours, the body temperature of AGRKO was significantly lower than that of wild type mice [79]. The histology of brown adipose tissue, however, showed that the lipid droplet size is smaller in the BAT of AGRKO than that of wild type mice [79]. Based on these observations, it was suggested that lipolytic activity was not affected by GR deletion in BAT upon cold exposure. The lipogenic activity of BAT in AGRKO mice upon cold exposure was not evaluated. Cold-induced plasma NEFA and ketone bodies levels were lower in AGRKO mice, for which the authors suggested that these phenotypes were due to the decreased lipolysis in WAT [79]. Another study found that glucocorticoid treatment increased the lipid accumulation in BAT of wild type but not AGRKO mice [45]. In agreement with this observation, another report found that glucocorticoid treatment elevated the expression of lipogenic genes in BAT of wild type mice. This effect was attenuated in AGRKO mice [46]. Notably, glucocorticoid treatment did not affect the expression of *Ucp1* and mitochondrial genes in BAT of wild type and AGRKO mice [45]. In BAT of wild type mice, glucocorticoid treatment enhanced whitening genes, such as *resistin* and *leptin*, which are usually highly expressed in white but not brown adipocytes. This effect is diminished in AGRKO mice [45]. Overall, GR regulates lipogenic and whitening genes instead of thermogenic genes in brown adipocytes to modulate brown adipocyte functions.

Glucocorticoids have also been shown to reduce the browning of white adipose tissue. Cold exposure activates the HPA axis, which increases the circulating adrenocorticotropic hormone (ACTH) and corticosterone in mice [95]. In primary adipocytes isolated from inguinal white adipose tissue, ACTH promoted the expression of Ucp1, which is attenuated by corticosterone treatment [95]. Glucocorticoids activated the transcription of miRNA, miR-27b, which targets *Prdm16* [30], which encodes a transcriptional coregulator that plays a key role in the differentiation of beige adipocytes. The reduction of Prdm16 expression in turn decreased the expression of Ucp1. Injecting lentivirus expressing antisense against miR-27b through tail vein effectively reduced the expression miR-27b in both subcutaneous and visceral adipose tissue [30]. This increased whole body energy expenditure and reduced adiposity in glucocorticoid-treated animals [30]. Glucocorticoids also activated another miRNA, miR-19b, which targets *β1 adrenergic receptor* (*Adrb1*), in primary adipocytes of subcutaneous white adipose tissues [31]. Overexpression of miR-19b reduced the expression of Ucp1 and oxygen consumption in adipocytes differentiated from precursor cells isolated from subcutaneous adipose tissues [31]. In contrast, reducing expression of miR-19b enhanced Ucp1 expression and oxygen consumption in these primary subcutaneous adipocytes [31]. Reducing miR-19b expression also blocked the ability of glucocorticoids to suppress Ucp1 expression and oxygen consumption[31]. Finally, while cold exposure induces beiging of subcutaneous white adipose tissues, warming (such as thermoneutrality, 30°C) “whitens” beige and brown adipose tissues [100, 101]. A recent study found that GR plays a critical role in the induction of whitening gene expression. Thus, compared to wild type mice when AGRKO mice were moved from 1 week 4°C treatment to 30°C for another week, the induction of whitening genes was delayed [102]. In contrast, mice put under cold exposure for 1 week and then injected with dexamethasone for another week while remaining at 4°C, displayed elevated expression of whitening genes but thermogenic gene expression was reduced [102].

Interestingly, injecting a synthetic glucocorticoid, prednisolone, into healthy humans during mild cold exposure (16–17°C) showed increased glucose uptake in BAT [32]. Moreover, in lean subjects, prednisolone treatment also increased supraclavicular skin temperature and energy expenditure in cold but not warm conditions [32]. These acute glucocorticoid responses were not observed in rodents. Glucocorticoid treatment increased isoprenaline-stimulated respiration and UCP1 expression in human primary brown adipocytes [32]. These results are also opposite to those found in murine primary brown and beige adipocytes. Thus, glucocorticoids exert species-specific effects on brown adipocyte functions. Another study found that including dexamethasone in the differentiation media of human primary brown adipocytes enhanced basal UCP1 expression and oxygen consumption rate [99]. However, isoprenaline-stimulated UCP1 expression and oxygen consumption rate were reduced by dexamethasone treatment [99]. In this study, 1 or 10 μM dexamethasone was used [99], whereas another study treated human primary brown adipocytes with cortisol for up to 1 μM [32]. Notably, dexamethasone is approximately 10 times more potent than cortisol. It is unclear whether the difference in ligands and doses used in the experiments resulted in these distinct observations.

## 7. Conclusion

Recent studies have made significant progress in establishing the role of adipocyte GR in whole body metabolic homeostasis. Nonetheless, many glucocorticoids and GR functions in adipocytes remain to be elucidated. For example, how do glucocorticoids and GR specifically promote central obesity in humans? What is the molecular basis of species-specific effects of glucocorticoids on brown adipocytes? One consistent observation in AGRKO mice is the role of adipocyte GR in the regulation of adipocyte lipolysis. Despite the identification of GR primary target genes involved in this process, the comprehensive understanding of this glucocorticoid regulated process, especially *in vivo*, requires more study. Moreover, does adipocyte GR play a role in specific physiological states that require the elevation of adipocyte lipolysis, such as exercise? Overall, with the availability of AGRKO mice and new methodology, such as CRISPR, we expect to learn more about glucocorticoids and GR function in adipocytes in the near future.

## Figures and Tables

**Table 1:** Comparison of Adipocyte GR Knock Out Mouse Studies.

Mouse Design	**Shen et al** [45]	Adipoq-Cre FVB/NJ background (Jackson Laboratory, 010803), GR floxed exon 3 [89] (Jackson Laboratory, B6.Cg-*Nr3cI*^*tml.lJda*^/J, 021021)
	**Bose et al** [46]	Adipoq-Cre FVB/NJ background (Jackson Laboratory, 010803), GR floxed exon3 [89]
	**Desarzens et al** [78]	Adipoq-Cre FVB/NJ background (Jackson Laboratory, 010803), GR floxed exon 3 [88]
	**Mueller et al** [91]	Adipoq-Cre FVB/NJ background (Jackson Laboratory, 010803), GR floxed exon 3 [88]
	**de Kloet et al** [86]	Adipoq-Cre C57BL/6 × 129 background. GR floxed exon 2 [90]
Fasting	**Shen et al** [45]	No change in fasting plasma insulin levels in both Chow and HFD. 4 hr fast showed no difference in plasma NEFA and glycerol but reduced lipolysis under isoproterenol stimulation
	**Bose et al** [46]	
	**Desarzens et al** [78]	
	**Mueller et al** [91]	AGRKO mice after 48hr fast. 2× higher body fat mass than controls. Increased fasting blood glucose. Impaired upregulation of fasting induced gene expression. AGRKO mice eWAT had decreased phosphorylation of HSL and perilipin. AGRKO had reduced cAMP generation, NEFA release.
	**de Kloet et al** [86]	
Chow	**Shen et al** [45]	No difference in body weight or composition. No difference in glucose tolerance or insulin sensitivity
	**Bose et al** [46]	No difference in bodyweight and fat mas
	**Desarzens et al** [78]	No difference in bodyweight, fat mass, or adipocyte size. No difference in plasma free fatty acids, and cholesterol. Slight decrease in TG levels in AGRKO mice. No difference in glucose and insulin levels
	**Mueller et al** [91]	4hr and 16 hr fasting blood glucose and plasma insulin levels showed no difference. No difference in GTT, but ITT showed delayed posthypoglycemic recovery likely due to decreased hepatic glucose production. Increased insulin induced AKT phosphorylation. Lower liver TG levels. Reduced NEFA release from 4hr and 16hr fasted AGRKO eWAT. Lower blood glucose levels during PTT in AGRKO mice
	**de Kloet et al** [86]	No difference in body weight, body mass, adiposity, and adipocyte morphology. NO difference in basal glucose levels. Unchanged adiponectin, insulin, and leptin in AGRKO mice
HFD	**Shen et al** [45]	14 week 58% Fat diet*: AGRKO trending to gain more weight. KO has decreased plasma TG and NEFA. Small increase in Gpat4 in eWAT. In liver, trending to decreased lipid metabolism and gluconeogenic genes with Mttp being significant. GTT and ITT no difference
	**Bose et al** [46]	16 weeks 42% Fat diet*: Similar body composition, BMD, BMC. No difference in glucose levels, GTT, nor ITT No difference in liver TG levels.
	**Desarzens et al** [78]	15 weeks 21.2% fat, 34.5% sucrose diet*: No difference in bodyweight, fat mass, or adipocyte size. No difference in plasma free fatty acids, triglycerides, and cholesterol. No difference in glucose and insulin levels. AGRKO mice on HFSD had increased area under the curve for GTT compared to fl/fl, which remained the same. Histology of adipose showed more crown-like structures in AGRKO mice, increased lipolcalin-2 and increased activation of JNK without effects on ERK1/2
	**Mueller et al** [91]	20 weeks 34.6% Fat diet* Reduced HFD induced obesity, decreased weight gain, reduced hepatic steatosis, improved glucose tolerance. Decreased FA and NEFA. Decreased expression of FA storage genes (Acaca, Gpat2, Dgat2, Pck1, Slc27a1 in eWAT. AGRKO mice had lower WAT weights than controls, No difference in plasma corticosterone levels, AGRKO mice had improved glucose tolerance and increased insulin sensitivity. AGRKO had decreased hepatic steatosis and decreased expression of Fasn, Gpat1, Pparq, Cd36, and Slc27a4 in liver.
	**de Kloet et al** [86]	40% fat diet*: AGRKO mice gain significantly less weight. Decreased adipose mass, decreased energy consumption.
Exogenous GC	**Shen et al** [45]	3mg/kg** Dexamethasone IP every other day for 2 months. No difference in body weight. Trend towards increased eWAT and iWAT. AGRKO mice more glucose and insulin tolerant than Flox mice. Improved insulin sensitivity in muscle. Decreased ability of Dex to induce whitening of BAT and Stimulated lipolysis.
	**Bose et al** [46]	10mg/kg** Dexamethasone for 2 weeks. No difference in blood glucose levels at baseline, GTT, or ITT. No difference in body composition, but AGRKO mice had higher fat mass than WT 10mg/kg Corticosterone 4 weeks: No difference in baseline blood glucose nor GTT and ITT. No difference in bodyweight, fat mass, BMD, nor liver TG levels
	**Desarzens et al** [78]	
	**Mueller et al** [91]	
	**de Kloet et al** [86]	0.1mg/kg** Dexamethasone injected AGRKO mice showed under stress an impaired negative feedback of HPA
BAT	**Shen et al** [45]	Cold exposure showed lower glycerol and NEFA levels in AGRKO mice.
	**Bose et al** [46]	10mg/kg acute Dexamethasone for 6 hrs resulted in upgregulation of some, but not all metabolism genes in AGRKO mice BAT.
	**Desarzens et al** [78]	
	**Mueller et al** [91]	4hr fast followed by 4h 4°C. Ability to maintain body temperature at 4°C was reduced. Lipid droplet decreased more in AGRKO BAT. Decreased cold induced thermogenesis due to impaired lipolysis and NEFA flux.
	**de Kloet et al** [86]	
HPA Axis	**Shen et al** [45]	
	**Bose et al** [46]	Dexamethasone suppression test showed decrease in AGRKO mice after Dexamethasone injection showing HPA axis activation was not altered.
	**Desarzens et al** [78]	
	**Mueller et al** [91]	
	**de Kloet et al** [86]	Similar baseline corticosterone levels. Under stress, AGRKO mice had increased corticosterone and hyperglycemia

Note:

* High fat diet use in the papers ranged from 21%–58% fat.

** Dexamethasone doses ranged from 0.1mg/kg to 3mg/kg and varied in duration from acute to chronic exposure. These high fat diet percentages as well as doses of dexamethasone could be responsible for the differences observed by the papers.
